# A zinc finger protein gene signature enables bladder cancer treatment stratification

**DOI:** 10.18632/aging.202984

**Published:** 2021-05-07

**Authors:** Jiandong Zhang, Chen Zhang, Peng Cao, Xiang Zheng, Baozhong Yu, Haoyuan Cao, Zihao Gao, Feilong Zhang, Jiyuan Wu, Huawei Cao, Changzhen Hao, Zejia Sun, Wei Wang

**Affiliations:** 1 Beijing Chaoyang Hospital Affiliated Capital Medical University, Beijing 100020, China; 2 Shanxi Bethune Hospital Affiliated Shanxi Academy of Medical Sciences, Taiyuan 030032, China; 3 State Key Laboratory of Membrane Biology, Institute of Zoology, Chinese Academy of Sciences and University of Chinese Academy of Sciences, Beijing 100101, China

**Keywords:** bladder cancer, zinc finger proteins, prognosis, treatment

## Abstract

Bladder cancer (BC) is a commonly occurring malignant tumor affecting the urinary tract. Zinc finger proteins (ZNFs) constitute the largest transcription factor family in the human genome and are therefore attractive biomarker candidates for BC prognosis. In this study, we profiled the expression of ZNFs in The Cancer Genome Atlas (TCGA) BC cohort and developed a novel prognostic signature based on 7 ZNF-coding genes. After external validation of the model in the GSE48276 dataset, we integrated the 7-ZNF-gene signature with patient clinicopathological data to construct a nomogram that forecasted 1-, 2-, and 3-year OS with good predictive accuracy. We then accessed The Genomics of Drug Sensitivity in Cancer database to predict the therapeutic drug responses of signature-defined high- and low-risk BC patients in the TCGA cohort. Greater sensitivity to chemotherapy was revealed in the low-risk group. Finally, we conducted gene set enrichment analysis of the signature genes and established, by applying the ESTIMATE algorithm, distinct correlations between the two risk groups and the presence of stromal and immune cell types in the tumor microenvironment. By allowing effective risk stratification of BC patients, our novel ZNF gene signature may enable tailoring more intensive treatment for high-risk patients.

## INTRODUCTION

Bladder cancer (BC) is one of the most common forms of malignant tumors affecting the urinary tract. Although there are many methods to diagnose and remove solid neoplasms, the high recurrence rate and mortality of BC are still not well controlled [1]. Therefore, there is an urgent need for effective diagnostic and prognostic biomarkers for BC. Although there are several treatment options, such as chemotherapy, for controlling the disease, there is still no effective guidance on how to choose chemotherapeutic drugs [2]. For this reason, strategies to individualize treatment are being explored actively. However, studies aimed at finding a reliable measure to stratify treatment for patients with BC have not yet yielded the desired results [3].

Zinc finger proteins (ZNFs) represent the largest transcription factor family in the human genome. These proteins are involved in diverse biological processes, including differentiation, development, metabolism, apoptosis, autophagy, and stemness maintenance. Over the last few decades, increasing evidence has supported key roles for ZNFs in cancer onset, progression, and metastasis [4]. For example, Chen et al. reported that ZNF830 promotes homologous recombination repair and survival of cancer cells in response to DNA damage, and that high expression of ZNF830 is associated with poor survival in lung and gastric cancer patients by mediating resistance to chemoradiotherapy [5]. Further examples include ZNF281 [6], which acts as an oncogene, ZNF185 [7] and ZNF750 [8], which serve as tumor suppressor genes, and ZEB1 [9] and ZBP89 [4], which appear to act as either oncogenes or tumor suppressor genes in different contexts. Interestingly, a recent study indicated that a single gene, encoding the zinc finger protein SPOP, can predict the prognosis of several tumors and guide stratification therapy [10].

**Figure 1:**
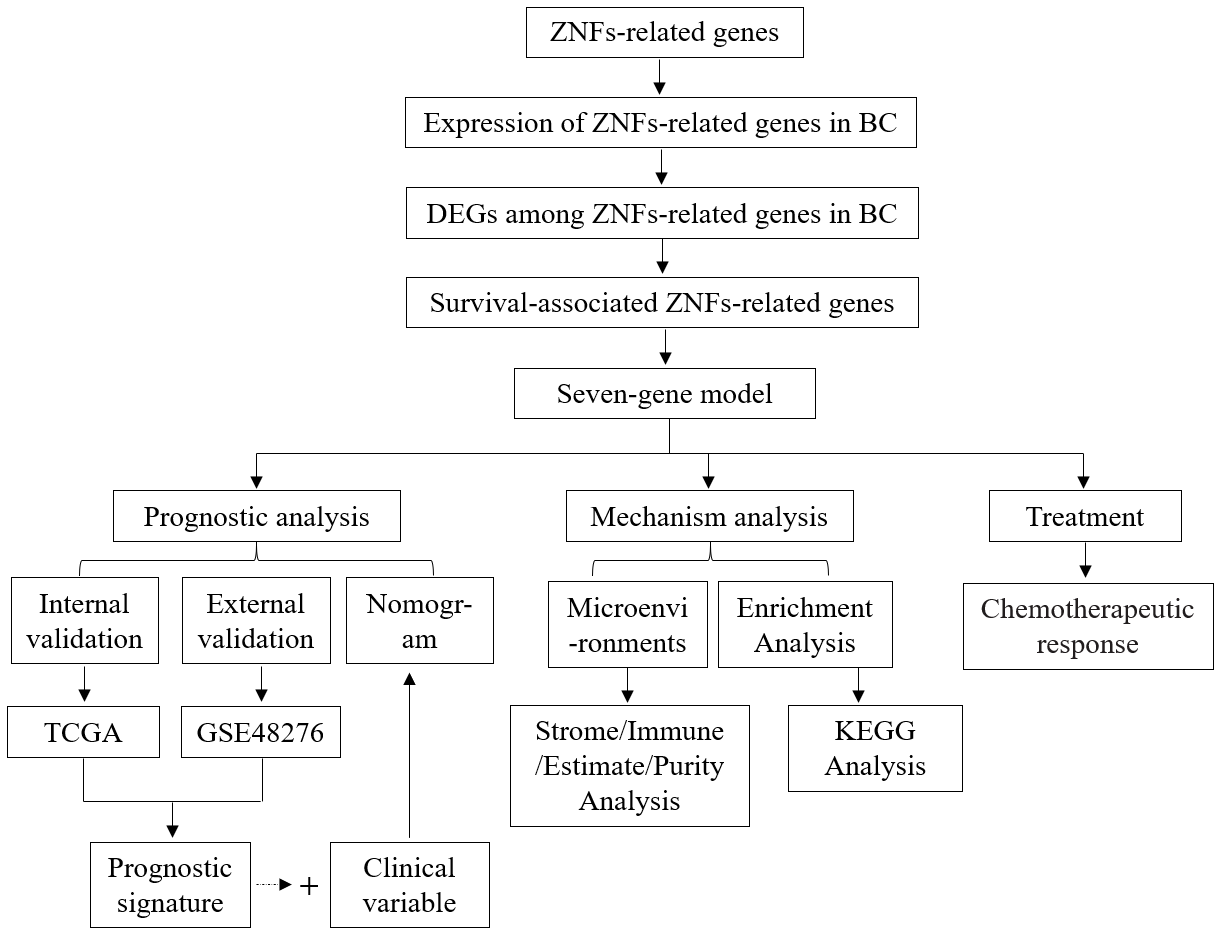
Flow chart of the study.

Identification of biomarker signatures represents a valuable approach to mine the wealth of information contained within biological samples [11, 12]. Since the significance of ZNFs in BC diagnosis, treatment, and prognosis remains unclear developing a biomarker signature based on ZNF protein genes might be helpful to guide decision-making to select appropriate treatments and to predict prognosis for BC patients. Moreover, the prognostic performance of the signature can be enhanced by constructing nomograms that integrate, along with the corresponding expression profiles, the patient’s clinical variables, e.g. tumor status [13]. Therefore, the goal of this study was to construct a ZNF gene-based signature to stratify patients, predict individual prognosis, and guide BC treatment. We also developed and tested a nomogram based on the ZNF-gene signature and clinical variables, assessed the signature’s association with stromal and immune cells in the tumor microenvironment and predicted, based on the expression of signature genes in low- and high-risk patients, their response to common chemotherapy agents. Our findings shed light on the potential contribution of ZNFs to the pathogenesis of BC and may inform clinical practice to guide individualized treatment. A flow chart depicting the analyses performed in this study is shown in Figure 1.

## RESULTS

### Identification of differentially expressed ZNF-coding genes in BC

**Figure 2:**
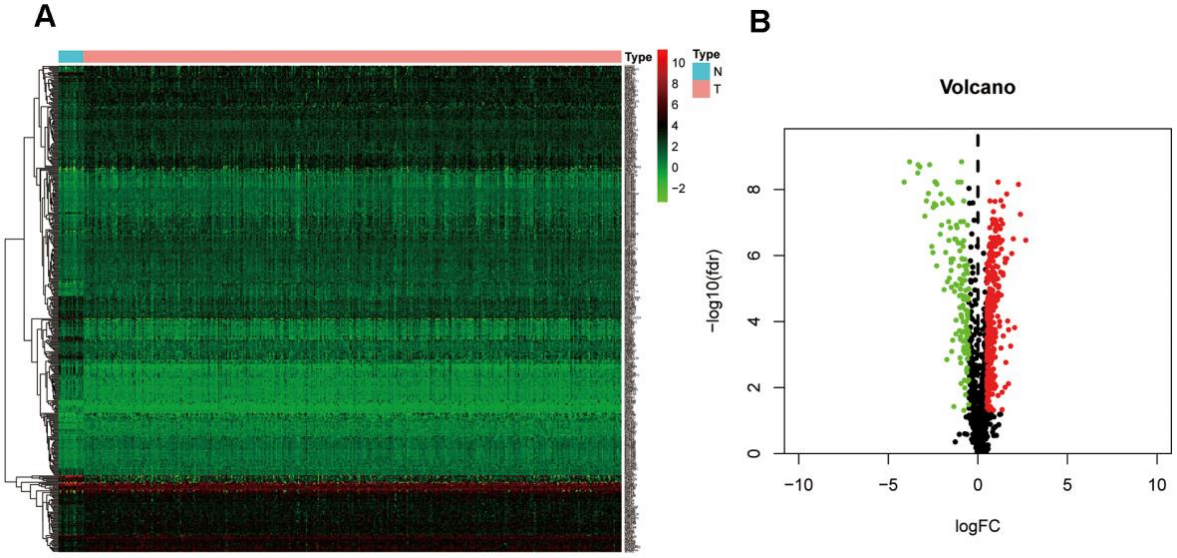
**Identification of differentially expressed ZNF genes in the TCGA-BC cohort.** (**A**) Heatmap depicting the expression levels of ZNF genes in tumor (T) and normal (N) samples. (**B**) Volcano plot representation of differentially expressed ZNF genes in the TCGA-BC cohort.

To determine the expression patterns of ZNF genes in BC, expression levels of 1818 human ZNF protein-coding genes retrieved from the UniProt database were evaluated in the transcriptional profiles of 403 muscle-invasive BC patients and 19 normal bladder controls, available in the TCGA (Figure 2A). A total of 319 upregulated and 139 downregulated ZNF-coding, differentially expressed genes (DEGs) were thus identified (Figure 2B and Supplementary Table 1).

### Development of a ZNF gene-based prognostic signature for BC

**Figure 3:**
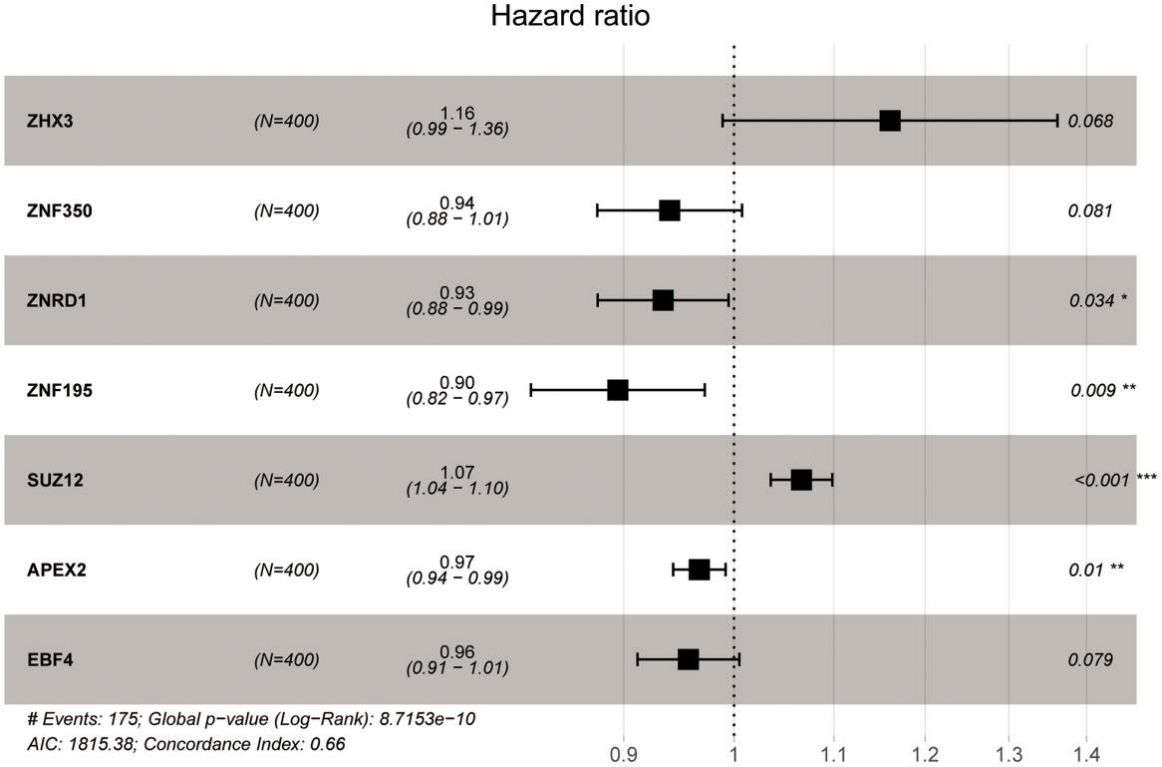
**Characteristics of BC-specific ZNF genes.** Forest plot showing hazard ratios (HRs) with 95% confidence interval (95% CI) of prognostic ZNF genes in BC based on multivariate Cox regression results.

A prognostic signature was then established by first identifying survival-associated ZNF-coding DEGs in the TCGA-BC cohort using univariate Cox regression. After screening out significant DEGs using LASSO regression and multivariate Cox regression, 7 BC-specific, prognostic ZNF genes were selected (Figure 3). After extracting the corresponding coefficient values (Table 1), individual risk scores were estimated based on coefficient-weighted expression levels of the selected genes.

**Table 1:** The coefficient of selected genes.

**Gene**	**coefficient**	**HR**	**HR.95Low**	**HR.95High**	***P* value**
ZHX3	0.148896	1.160553	0.989119	1.361699	0.067879
ZNF350	-0.0615	0.940356	0.877571	1.007633	0.08111
ZNRD1	-0.06775	0.934497	0.877895	0.994749	0.033577
ZNF195	-0.11088	0.895049	0.823812	0.972446	0.008786
SUZ12	0.064369	1.066486	1.035628	1.098264	1.73E-05
APEX2	-0.03312	0.967418	0.943455	0.99199	0.009642
EBF4	-0.04355	0.957387	0.912023	1.005007	0.078698

**Figure 4:**
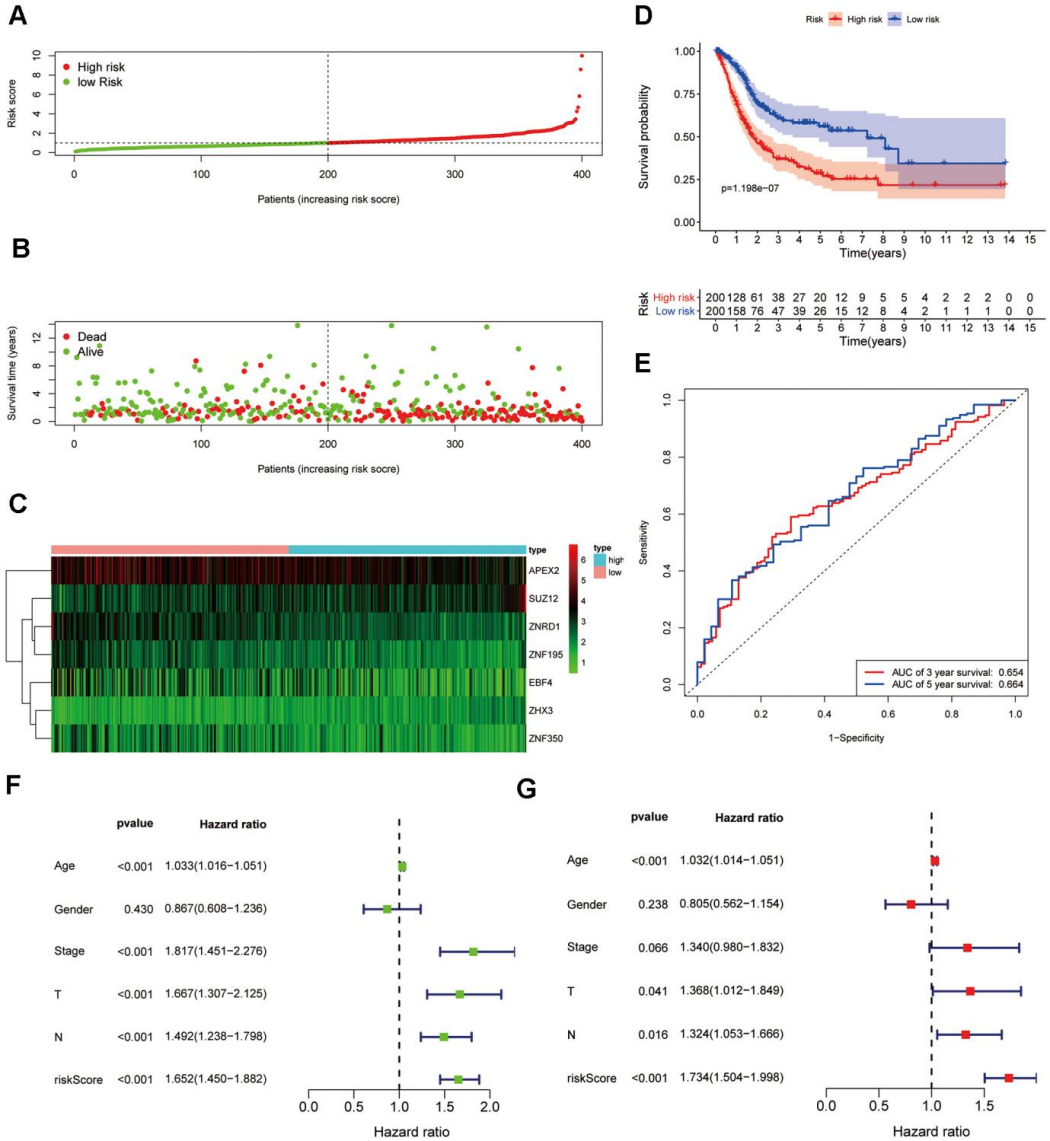
**Development of a prognostic signature for BC based on 7 ZNF genes.** (**A**) Distribution and median value of the risk scores in the TCGA cohort. (**B**) Survival status of patients in the different risk groups. (**C**) Heatmap of the expression profiles of the 7 ZNF genes included in the prognostic signature. (**D**) Time-dependent ROC curve of the 7-gene signature. (**E**) Survival analysis of the signature-defined risk groups. (**F**) Univariable and (**G**) multivariable analyses adjusting for risk score, age, gender, tumor stage, and TNM.

Following exclusion of three BC patients with no follow-up information in the TCGA cohort, the patients were stratified into a high-risk group (n=200) and a low-risk group (n=200) according to the median cut-off value (Figure 4A). As shown in Figure 4B, patients with high risk had a higher probability of early death than those with low risk. Consistently, the heatmap of expression profiles in the TCGA dataset showed distinct differences between groups (Figure 4C). Moreover, survival analysis indicated that patients in the high-risk group had a significantly worse OS than their low-risk counterparts (Figure 4D, *P* <0.001). The ZNF gene-based prognostic signature showed good performance, with AUCs of 0.654 and 0.664 at 3- and 5-year follow-up respectively (Figure 4E). Furthermore, the signature was significantly predictive of survival on univariable (Figure 3) and multivariable (Figure 3) analyses that included risk score, age, gender, tumor stage, and TNM (*P*<0.001 for all variables, except gender and tumor stage).

### External validation of the prognostic signature

**Figure 5:**
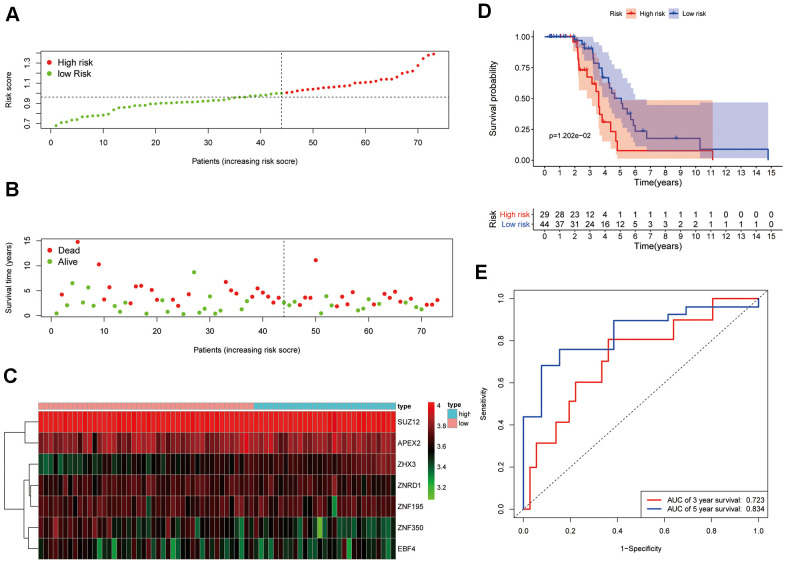
**Validation of the 7-ZNF-gene prognostic signature in a GEO dataset.** (**A**) Distribution and median value of the risk scores in the GSE48276 cohort. (**B**) Survival status of low-risk and high-risk patients. (**C**) Heatmap of the expression profiles of the 7-ZNF-gene signature. (**D**) Time-dependent ROC curve of the prognostic signature. (**E**) Survival analysis of signature-defined risk groups.

To verify the predictive value of our model, samples from patients in the GSE48276 cohort served as external testing data after categorizing them into high- or low-risk groups using the median value calculated with the same formula and cutoff value applied before (Figure 5A). The expression profiles corresponding to the signature genes are shown in Figure 5C. Similar to the results obtained in the original TCGA cohort, patients in the high-risk group were more likely to die earlier (Figure 5B) and had a reduced survival time compared with those in the low-risk group (Figure 5D; *P*= 0.001). Moreover, the prognostic performance of the ZNF gene-based prognostic signature showed acceptable discrimination, with AUCs of 0.723 and 0.834 at 3- and 5-year follow-up, respectively (Figure 5E).

### Predictive accuracy of a ZNF gene-based nomogram

**Figure 6:**
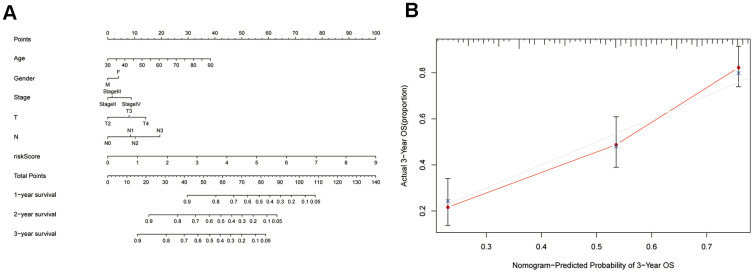
**Construction of a nomogram based on the 7-ZNF-gene signature.** (**A**) Nomogram based on the ZNF-gene signature and clinical information. (**B**) Decision curve analysis evaluating the clinical utility of the nomogram at 3-year survival.

After asserting the prognostic reliability of the 7-ZNF-gene signature on BC outcomes, we used it along with patient clinicopathological data to construct a nomogram to forecast 1-, 2-, and 3-year OS (Figure 6A). The calibration plot of the nomogram indicated optimal predictive accuracy, with a close overlap between predicted and actual survival rate (Figure 6B).

### The ZNF-gene signature predicts differences in BC microenvironment

**Figure 7:**
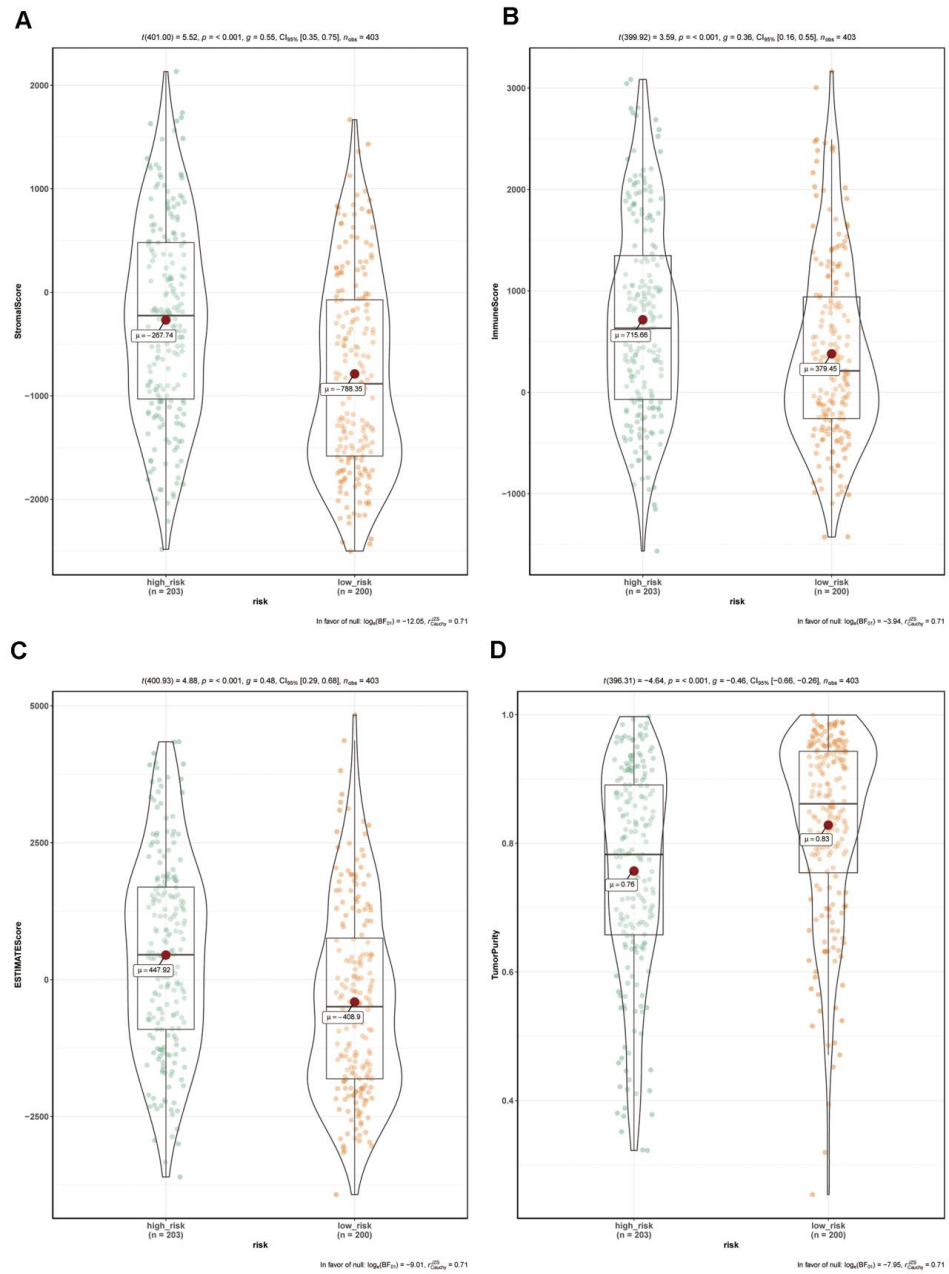
**Comparison of tumor microenvironment composition between risk groups in the TCGA-BC cohort.** (**A**) Comparison of stromal scores between risk groups (*P*<0.001). (**B**) Comparison of immune scores between risk groups (*P*<0.001). (**C**) Comparison of ESTIMATE scores between risk groups (*P*<0.001). (**D**) Comparison of tumor purity between risk groups (*P*<0.001).

To assess whether the ZNF-gene signature can help distinguish differences in the tumor microenvironment of BC, we employed the ESTIMATE tool to compare gene expression signatures of stromal and immune cells among risk groups. The stromal score ranged from -788.35 to -267.74 (Figure 7A), the immune score ranged from 379.45 to 715.66 (Figure 7B), and the ESTIMATE score ranged from -408.9 to 447.92 (Figure 7C), with statistically significant differences (*P*<0.001 for all scores) detected between the high-risk and low-risk groups. Meanwhile, a lower tumor purity, distributed between 0.76 and 0.83 (Figure 7D) was observed in the high-risk group (*P*<0.001).

### Correlation between tumor-infiltrating immune cells and the ZNF-gene signature

**Figure 8:**
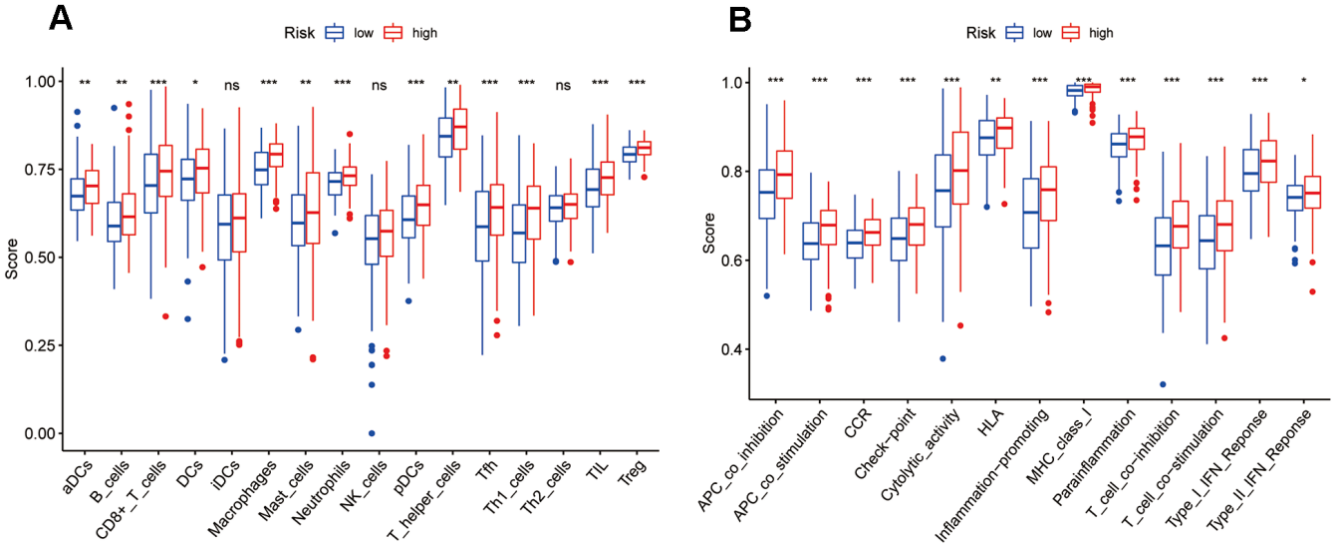
**Comparison of ssGSEA scores between risk groups in the TCGA-BC cohort.** (**A**) Scores of 16 immune cell types. (**B**) Functions enriched in the 7-ZNF-gene signature. CCR, cytokine-cytokine receptor; ns, not significant; *, *P*< 0.05; **, *P*< 0.01; ***, *P*< 0.001 (adjusted P values).

To further investigate the relationship between the ZNF gene signature’s risk score and the tumor’s immune status, the enrichment scores of diverse immune cell subpopulations, and their related functions or pathways, were quantified in the TCGA-BC cohort using ssGSEA. The results showed that scores for cell types related to the antigen presentation process, including dendritic cells (DCs), activated DCs, plasmacytoid DCs, tumor-infiltrating lymphocytes (TILs), B cells, macrophages, mast cells, neutrophils, CD8 T cells, T helper (Th) cells, Th1 cells, T follicular helper cells (Tfhs), and regulatory T cells (TRegs) were significantly different between risk groups (adjusted *P* < 0.05 for all; Figure 8A). On KEGG analysis, cytokine-cytokine receptor interaction showed a higher score in the high-risk group (adjusted *P* < 0.05; Figure 8B). Moreover, the high-risk group showed also enrichment in the activity of checkpoint molecules and higher scores for macrophages or Tregs, whereas scores for type II IFN response, type I IFN response, and NK cells were instead lower (adjusted *P*< 0.05, Figure 8A, 8B).

### The ZNF-gene signature predicts chemotherapy response in BC

**Figure 9:**
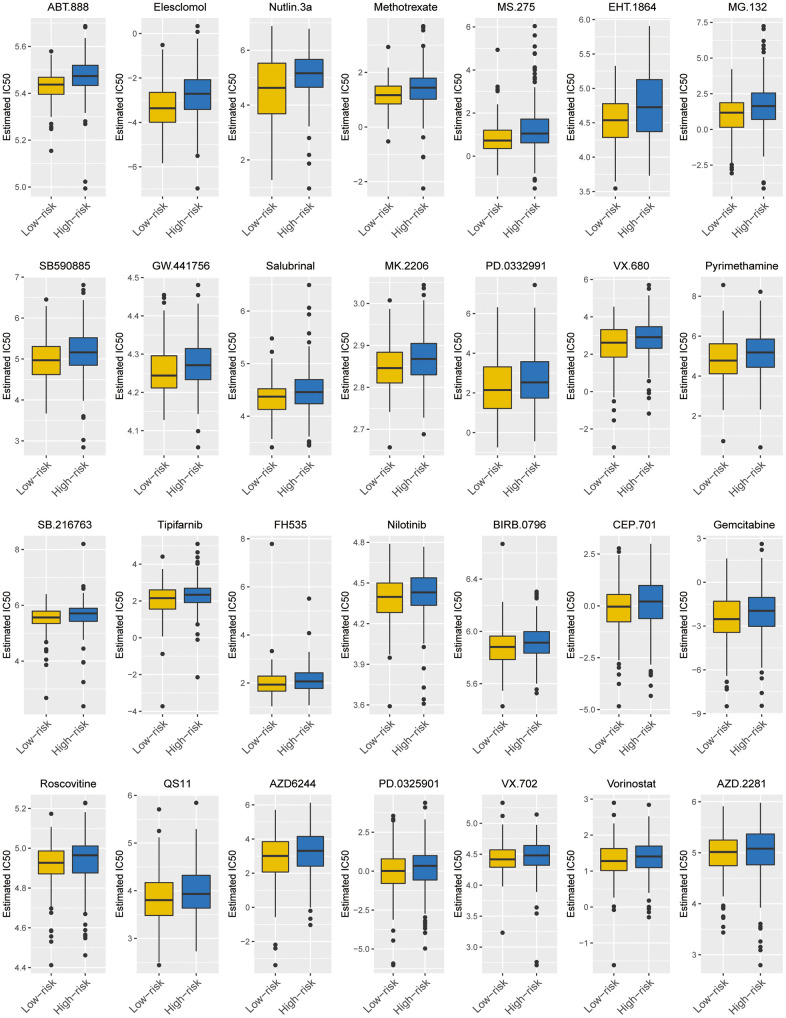
**Predicted responses to chemotherapy for risk groups in the TCGA-BC cohort.** Boxplots exhibiting the estimated IC50 values of 28/138 screened drugs for tumors cells from the two risk groups (*P*<0.05 for all).

Considering that chemotherapy is still the most effective adjuvant measure to treat BC, we accessed the Genomics of Drug Sensitivity in Cancer (GDSC) database to estimate the response of low-risk and high-risk BC patients to commonly used drugs. The correlation between risk groups and IC50 values for 138 chemotherapeutic agents was visualized using scatterplots. We found significant discrimination between groups in the estimated IC50 values of 28 drugs (Figure 9, *P*< 0.05 for all). Hence, we concluded that the low-risk group may be more sensitive to common chemotherapies during clinical treatment.

### Association of the ZNF signature genes with BC progression

**Figure 10:**
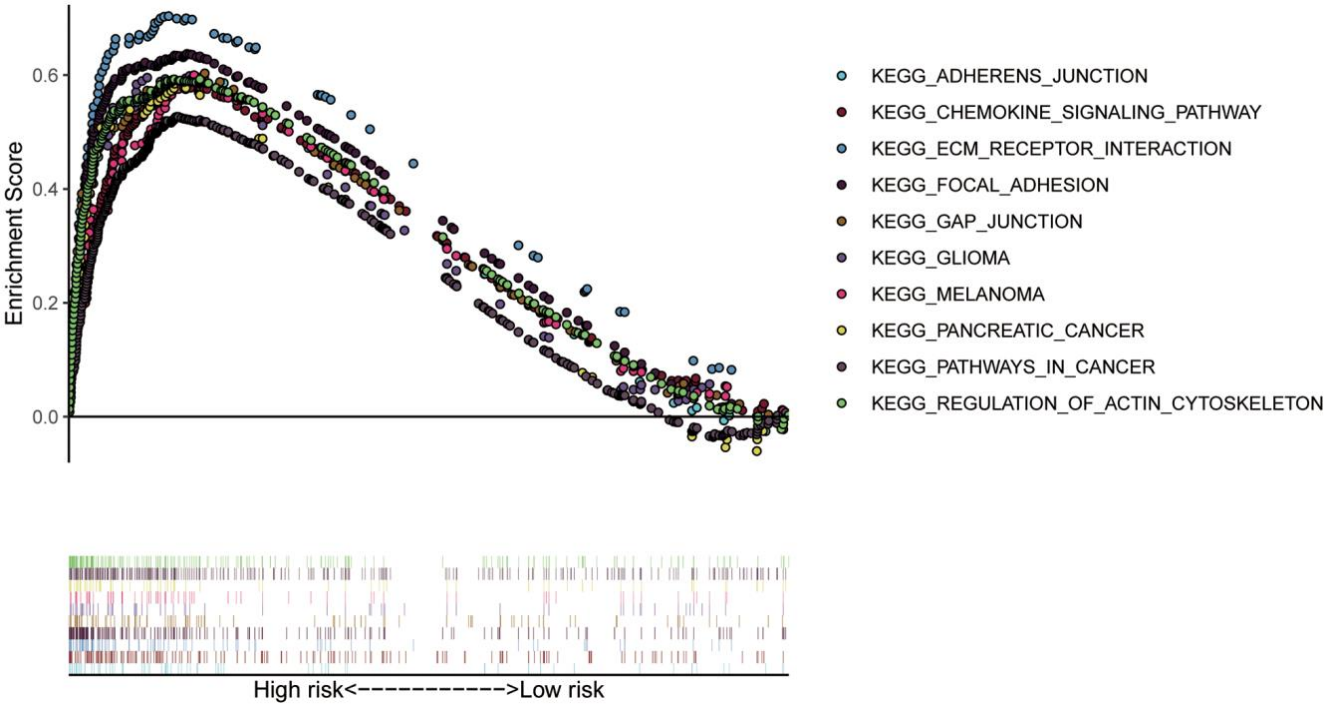
**KEGG pathway enrichment analysis of signature genes.** ECM, extracellular matrix.

To gain insight into the functions of the 7 ZNF protein-coding genes included in our signature, we performed KEGG enrichment analysis based on GSEA enrichment scores. The results indicated that the expression patterns that conformed to the high-risk group were enriched in KEGG terms related to tumor progression, such as extracellular matrix (ECM)-receptor interaction, adherens junction, chemokine signaling pathway, and gap junction (Figure 10, P< 0.05 for all). Interestingly, our ZNF-gene signature was closely correlated with other malignancies, for instance melanoma, pancreatic cancer, and glioma. These results suggest that the ZNF protein genes comprising our BC signature may also drive the onset or progression of other types of cancers.

## DISCUSSION

BC, a common urinary malignancy, is associated with poor prognosis in advanced stages. Thus, identification of novel biomarkers will help assess prognosis, screen out patients in need of systemic therapy, and guide individual treatment. ZNFs represent highly promising biomarkers for BC. First, they are one of the most abundant groups of proteins and have a wide range of molecular functions. Second, ZNFs are involved in tumorigenesis, cancer progression, and metastasis formation. Third, ZNFs may act as oncogenes or tumor suppressors and thus serve as valuable prognostic factors. Fourth, ZNF protein genes are closely involved in the oncogenesis of BC. For instance, activation of MDM2, a ZNF domain-containing E3 ubiquitin ligase, leads to ubiquitylation and proteasomal degradation of p53, a major tumor suppressor protein closely involved in the occurrence, progression, and metastasis of BC [14]. Furthermore, ZNFs’ genes are involved in telomere maintenance and genome integrity in cancer and aging [15]. Therefore, in this study we conducted bioinformatics analysis to explore whether differential expression of ZNF genes could be exploited to predict BC outcomes and aid risk stratification.

Based on transcriptional data from the TCGA-BC cohort, we identified 7 prognostic ZNF-coding genes that may serve as valuable biomarkers in the clinical setting. Similar to other analyses [16, 17], the prognostic signature based on the 7 ZNF genes categorized BC patients into two subgroups with different survival outcomes. Importantly, the ability of the gene signature to distinguish high-and low-risk patients, and to estimate OS, was independently validated in the GSE48276 dataset.

The increasing use of nomograms, based on integrated analysis of tumor signatures (gene expression) and patient-specific clinicopathological data, has the potential to drastically improve disease prognostication [13]. Based on the 7-ZNF-gene prognostic signature, we constructed a nomogram that showed high predictive accuracy for OS. Moreover, besides predicting survival outcomes, the ZNF-based prognostic signature also predicted differences in the composition of the tumor microenvironment, determined by differential representation of stromal and immune cells. In addition, by inputting the ZNF-gene signature profiles of the BC risk groups into the GDSC database, we found that patients in the low-risk group may be more sensitive to common chemotherapies during clinical treatment.

Our ZNF protein gene-based signature included 7 genes, i.e., ZHX3, ZNF350, ZNRD1, ZNF195, SUZ12, APEX2, and EBF4. Among these, 4 genes (ZHX3, ZNF350, ZNF195, and SUZ12) have been implicated, as discussed below, in the onset and progression of BC. Two signature genes (ZHX3 and SUZ12) were positively correlated with the occurrence of BC, while a negative correlation was instead determined for the remaining 5 genes (ZNF350, ZNRD1, ZNF195, APEX2, and EBF4). In line with a recent study [18], our results showed that ZHX3 plays an oncogenic role in BC pathogenesis. Interestingly, ZHX3 has also shown to act as a tumor suppressor in renal cell carcinoma [19], breast cancer [20], and liver cancer [21]. A role for SUZ12 overexpression in BC is also supported by previous studies. Fan et al. showed that SUZ12 overexpression promoted BC progression by stimulating colony formation, migration, and invasiveness of BC cells [22]. In turn, Lee at al. reported a gene signature that includes E2F1-EZH2-SUZ12 and shows predictive value for prognosis in BC [23]. All these data strongly indicate that ZHX3 and SUZ12 act as oncogenes in BC, and suggest that a signature based on these two genes may be of significance to guide patients’ treatment and improve prognosis.

The 5 protective genes included in our signature were further retrieved and analyzed. Our results showed that ZNF350 expression was associated with reduced BC risk, which is consistent with previous results [24]. Similarly, another report associated high expression levels of ZNF195 with favorable survival in BC [25]. In contrast, the roles of ZNRD1, APEX2, and EBF4 in BC onset and development had not, to our knowledge, been as yet explored. Interestingly, upregulated expression of zinc ribbon domain containing 1 antisense RNA 1 (ZNRD1-AS1), a negative regulator of ZNRD1, was detected in BC [26]. This finding indirectly supports the reduction in ZNRD1 expression detected by our analysis. The protein encoded by APEX2 plays an important role in both nuclear and mitochondrial base excision repair [27]. Our findings on APEX2 are supported by evidence that this ZNF protein serves as a synthetic lethal target in BRCA1- and BRCA2-deficient colonic and ovarian cancer cell lines [28]. In contrast, APEX2 overexpression has been reported in liver cancer [29] and myeloma [30]. Jensen et al. recently explored the expression of APEX2 in multiple cancers and indicated that this gene possesses tissue-specific characteristics [31]. Little research has been done on the role of EBF4 in cancer [32, 33], and a few evidences suggest that it plays important roles in neural development and B-cell maturation [34]. Based on current knowledge, and pending further investigation, our findings suggest that the 7 genes included in our signature may exert important roles in the tumorigenesis and progression of BC.

The ZNF-gene signature identified by us showed a close association with the tumor microenvironment, as it predicted differential representation of stromal and immune infiltrating cells among risk groups. These cells form the major fraction of non-tumor cells in tumor tissues and establish key interactions that influence growth, survival, and metastasis of tumor cells [35]. Based on expression patterns of our ZNF-gene signature among risk groups, higher stromal, immune, and ESTIMATE scores, as well as lower tumor purity, were calculated for the high-risk group. The presence of distinct subsets of immune cells within the tumor microenvironment does not only influence tumor progression, but impact treatment responses as well. Our results showed that tumor-infiltrating immune cell populations were more abundant in the high-risk than in the low-risk group. In particular, the representation of immune cell types involved in antigen presentation was significantly greater in the high-risk group. A mechanistic explanation of this phenomenon may involve a distinct effect of ZNFs on the expression of chemokines, leading to enhanced recruitment of tumor-infiltrating cells.

Our ZNF-gene signature for BC was able to predict chemotherapy sensitivity and may thus help guide treatment selection. Although there are many chemotherapeutic options for BC treatment, there are so far no consensual guidelines in this regard. GDSC is the largest public database containing information on drug sensitivity of cancer cell lines and molecular markers of drug response based on large genomic data. Here, we built statistical models based on gene expression and drug sensitivity data derived from BC cell lines. Our findings showed that the IC50 of 28 chemotherapeutic agents, including gemcitabine and methotrexate [36], predicted using the GDSC dataset, were lower for the low-risk group. While these data suggest that low-risk BC patients are more sensitive to chemotherapy, additional analyses implementing new tools like CancerTracer, which allows further assessment of intratumor heterogeneity, will further help guide chemotherapy drug selection for personalized treatment [37].

In light of evidence that links deregulation of ZNFs’ expression with either pro-oncogenic or tumor-suppressing activities, the significance of ZNFs in cancer tumorigenesis, progression, and metastasis is a topic of intense research. To our knowledge, our study is the first to document a full ZNF gene-based signature with prognostic ability in BC. Nevertheless, there are multiple limitations to the present study. First, since matched, normal bladder samples were far fewer than the BC specimens analyzed, the results need to be verified by expanding the number of controls. Second, the functional relationship between the ZNF gene signature members and non-tumor cells in the tumor microenvironment, especially infiltrating immune cells, could not be elucidated and requires future *in vitro* and *in vivo* studies.

In summary, we established a novel ZNF gene-based prognostic signature that divides BC patients into two subgroups with different survival outcomes and constructed a nomogram to help clinical decision-makers provide optimal treatment. The prognostic signature is associated with differences in stromal and immune cell components of the tumor microenvironment, and predicts sensitivity to chemotherapeutic agents in low risk and high risk BC patients. Our study may stimulate further research on the role of ZNFs on BC and help guide stratified therapy to provide individualized treatment.

## MATERIALS AND METHODS

### Sample information and data collection

The transcriptional data and corresponding clinical information of 403 chemotherapy-naïve BC patients and 19 normal bladder control samples were downloaded from the TCGA website (https://www.cancer.gov/tcga.) [38]. Gene expression profiles were normalized by the “limma” R package. The GSE48276 [39] dataset, containing mRNA expression profiles from 73 BC tissues, was downloaded from the GEO database (https://www.ncbi.nlm.nih.gov/geo/) and used as external validation data. By accessing the UniProt [40] website (http://www.uniprot.org), we retrieved the latest list of ZNF-coding genes, which includes 1818 genes.

### Construction and validation of a prognostic model

Differentially expressed genes (DEGs) between tumor and matched normal tissues were identified in the TCGA cohort by the “limma” R package using a false discovery rate (FDR) < 0.05. ZNF-coding genes with prognostic value were screened out by univariate Cox analysis of overall survival (OS) and p values were adjusted by Wilcoxon tests. To minimize the risk of overfitting, a prognostic model was constructed using LASSO penalized Cox regression analysis [41]. Variable selection and shrinkage of the prognostic model were achieved by running the LASSO algorithm in the “glmnet” R package. The independent variables of the model were the DEGs with prognostic values, and the response variables were OS and status of patients in the TCGA cohort. To improve the reliability and objectivity of the results, 1000 cross-validation runs were performed to determine the optimal value of the penalty parameter (λ). The normalized expression level of each gene and its corresponding regression coefficient were used to calculate the risk score of patients. The formula was established as follows: score = esum (each gene’s expression × corresponding coefficient). The patients were stratified into high-risk and low-risk groups according to the median value of the risk score. To evaluate the predictive power of the gene signature, a time-dependent ROC curve was built with the “survivalROC” R package. Clinical characteristics, including age, gender, stage, and tumor-node- metastasis (TNM) status were collected from TCGA database. Univariable and multivariate Cox regression analysis were run using clinical data and risk scores to determine whether the predictive value of the risk scores was independent of the clinical characteristics. P< 0.05 was considered statistically significant.

The prognostic signature, with an identical risk score formula and threshold, was then verified against the BC dataset GSE48276. Performance of the prognostic model on the validating dataset was represented via risk score-based plots depicting prognostic gene expression, risk score distribution, and survival status among patients.

### Construction of a ZNF-based nomogram

A nomogram combining the risk score model derived from the prognostic ZNF signature and clinicopathological factors was constructed using the “rms” R package. Discrimination of the nomogram was verified using ROC analysis at 1-, 2-, and 3-year follow-up, and predictive accuracy was assessed through a calibration plot contrasting predicted vs actual survival.

### Estimation of stromal and immune scores

Stromal and immune cells play a fundamental role in shaping the tumor microenvironment [42]. To further confirm the predictive power of our prognostic signature on tumor progression, the ESTIMATE algorithm in R was used to assign stromal and immune scores to the high-risk and low-risk groups defined by the model. The ESTIMATE score, reflecting tumor purity, was thereby derived [35].

### Analysis of tumor-infiltrating immune cells

The “clusterProfiler” R package was employed to perform Kyoto Encyclopedia of Genes and Genomes (KEGG) analyses based on the DEGs (|log2FC| ≥ 1, FDR < 0.05) between the high-risk and low-risk groups. P values were adjusted with the Wilcoxon test. Tumor infiltration scores for 16 immune cell types and activation status for 13 immune-related pathways were assessed with the single-sample gene set enrichment analysis (ssGSEA) function [43] in the “gsva” R package.

### Prediction of chemotherapeutic response

The chemotherapeutic response of each BC patient in the TCGA cohort was predicted according to the public pharmacogenomic database Genomics of Drug Sensitivity in Cancer (GDSC, https://www.cancerrxgene.org/). The GDSC database contains data from a large collection of human cancer cell lines, anticancer compounds, and experimental data on drug sensitivity [44]. The prediction of drug sensitivity (IC50) values was conducted using the R package “pRRophetic” [45], which uses a ridge regression model based on cancer cell lines’ expression profiles in the GDSC.

## Supplementary Material

Supplementary Table 1
